# Milk Potential of Pantaneira Cows, a Local Breed, at Organic System

**DOI:** 10.3390/ani10061079

**Published:** 2020-06-23

**Authors:** Willian Biazolli, Marcus Vinicius Morais de Oliveira, Dirce Ferreira Luz, Leonardo de Oliveira Seno, Alysson Martins Wanderley, Pedro Gustavo Loesia Lima, Tatiane Fernandes, Fernando Miranda de Vargas Junior

**Affiliations:** 1 Programa de Pós-Graduação em Zootecnia, Universidade Federal da Grande Dourados, Dourados, MS 79804-970, Brazil; willian.biazolli@gmail.com (W.B.); marcusvmo@yahoo.com.br (M.V.M.d.O.); leonardoseno@ufgd.edu.br (L.d.O.S.); tati-_-tati@hotmail.com (T.F.); 2 Programa de Pós-graduação em Zootecnia, Universidade Estadual de Mato Grosso do Sul, UEMS, Aquidauana, MS 79200-00, Brazil; alyssonmw@yahoo.com.br (A.M.W.); pedroloesia@hotmail.com (P.G.L.L.); 3 Curso de Ciências Biológicas, Universidade Federal de Mato Grosso do Sul, UFMS, Aquidauana, MS 79200-00, Brazil; Dirceluz@yahoo.com.br

**Keywords:** animal genetic resource, conservation, dairy, Pantanal, cattle

## Abstract

**Simple Summary:**

Pantaneira cattle are descendants of the genetic group of crossed European animals and are a breed locally adapted to the Brazilian Pantanal. The use of this breed in organic systems can have benefits for the conservation of the breed and because it is a genetically rustic breed. The aim of this study was to evaluate the performance of Pantaneira primiparous cows under systems with reduced use of concentrate, simulating organic production conditions. There was a reduction in milk yield, but the energy-corrected milk yield and efficiency were not affected. The Pantaneira breed has the genetic potential for the maintenance of competitive production and quality in organic systems.

**Abstract:**

Pantaneiro cattle (*Bos taurus taurus*) is a breed locally adapted to the Brazilian Pantanal. Local breeds are essential for the quality production of organic systems based on planned grazing practices, because of their results in resilient and productive ecosystems, enhancing biodiversity. This study aimed to evaluate the performance of Pantaneira primiparous cows, and systems with reduced use of concentrate, simulating organic production conditions. Five animals, with an average body weight of 396.2 ± 43.5 kg, were kept in individual continuous grazing regimes and supplemented with different concentrate levels (1.2%, 0.9%, 0.6%, 0.3%, and 0.0% of body weight). The animals were allocated at random in a 5 × 5 Latin square design repeated twice during the study time. The cows had a low dry matter and nutrient intake with a reduction in concentrate level, with improvement in neutral detergent fiber digestibility and a reduction in total nutrient digestibility. No changes were observed in plasma glucose levels or urea excretion, but the plasma urea nitrogen decreased with reductions in concentrate levels. There was a reduction in milk yield, but the energy-corrected milk was not affected by the reduction in concentrate levels; furthermore, the milk yield efficiency was not affected. The milk fat content improved with the reduction in concentrate levels. The Pantaneira breed has the genetic potential for the maintenance of competitive production and quality in organic systems.

## 1. Introduction

Consumer demand for high quality and safe animal products, such as organic products, has been growing. Organic foods are commonly marketed as being healthy foods from healthier animals [1]. Local breeds are essential for the maintenance of the competitive production and quality of organic systems [2]. However, the use of highly specialized breeds has led to a decline in agricultural animal biodiversity [3]; the rate of extinction of known races and those who have not had their genetic potential investigated is alarming [4].

Pantaneira cattle are descendants of the crossing of European animals, carriers of the genes of *Bos taurus taurus* [5], and are among the select group of local breeds considered Brazilian. According to an FAO report [6], there are currently only 500 pure-bred animals, split between two conservationist herds, because the main threat to Pantaneira breed is the crossing with commercial breeds. Pantaneira cattle have a smaller body size but have kept their bovine ancestors’ genes related to high maternal ability and longevity [7] and featuring high birth rate, normal deliveries, and healthy offspring [8]. Another important characteristic is the presence of the G1 allele of the bovine growth hormone gene (thrifty gene), which has essentially become extinct in commercial breeds [9] but can be found in Pantaneira cattle [6]. It is also the only breed of cattle that can graze pastures under water [10] and can spend months in extreme drought or in flooded environments without presenting problems to the hooves or suffer nutritional harm. This is a result of it being a genetically rustic breed, adapted to exposure to pathogens such as trypanosomiasis, myiasis, worms, and ticks [11] and also high temperatures and different types of food [12].

Due to its rusticity and ability to use the food produced in the Pantanal region, the Pantaneira breed has the potential to be used in organic production. Since organic standards recommend the use of local breeds, organic systems are characterized by a stronger dependence on local food resources, a higher proportion of feeding systems based on pastures, and with restricted amounts of concentrated feeds and medical drugs [2].

In this context, the Pantaneira breed becomes an interesting option for livestock breeding, especially for small producers, in organic systems. Thus, the determination of performance indices and the evaluation of potential dairy grazing conditions with reduced concentrate supplementation are important in the generation of new approaches to the production of milk from this breed and its physical and chemical composition. This study aimed to evaluate the performance of Pantaneira cows under systems with reduced use of concentrate, simulating organic production conditions, and, from the perspective of sustainable management in small farms, to help in the conservation of natural areas of Pantanal, contributing to better environmental conditions and ensuring animal genetic biodiversity.

## 2. Materials and Methods

The study was conducted at the Pantaneira Bovine Conservation Center (NUBOPAN) of the State University of Mato Grosso do Sul (UEMS) of the city Aquidauana, in the region of the High Pantanal, Sul-Mato-Grossense (Brazilian wetlands in Mato Grosso do Sul State), Brazil, whose geographical coordinates are 20°2’ S; 55°48’ W and altitude is 149 m. The climate, according to the Köppen climate classification [13], is tropical savanna with dry winters. During the testing period, the rainy season (October to February), an accumulated rainfall of 225 mm was observed, with maximum and minimum values of 37.1 and 24.9 °C for temperature and 92.1 and 38.9% for relative humidity, respectively. All procedures used herein were conducted with approval and supervision of the Ethical Committee of the Universidade Estadual de Mato Grosso do Sul (Protocol: 036/2019).

The experimental design used was Latin square, formed by five primiparous Pantaneira cows; fed with five supplementation levels, ranging from 1.2% of live weight, which corresponds with what is practiced in conventional farms, to zero, corresponding to pasture-based production or organic systems; at five periods of 14 days; with two replicates throughout the duration. The cows, with an average body weight (BW) of 396.2 ± 43.5 kg and 42 months old, were kept in an individual grazing regime in five paddocks of *Panicum maximum* cv. Mombasa. The treatments corresponded to supplementation with 1.2% (5.28 kg/d), 0.9% (3.96 kg/d), 0.6% (2.64 kg/d), 0.3% (1.32 kg/d), and 0.0% BW of concentrated feed. The concentrate was prepared with corn grain (59.5%), soybean meal (39.5%), and minerals (1%; warranted levels per kg of calcium: 120 g, cobalt: 55 mg, copper: 1530 mg, sulfur: 12 g, phosphorus: 88 g, iodine: 75 mg, manganese: 1300 mg, selenium: 15 mg, sodium: 132 g, and zinc: 3630 mg). The chemical composition of the concentrate was crude protein: 22.11% of dry matter (DM), rumen degradable protein: 48.02% of DM, total digestible nutrients: 80.04% of DM, and digestible energy: 3.53 Mcal/kg of DM. The concentrate was offered to the cows in individual troughs in two equal portions after milking, and the cows were led to the pastures so that each paddock was grazed in a continuous stocking, with the target height at the beginning of grazing of 90 cm.

Each animal paddock contained 0.5 hectares of forage area, a water cooler, a mineral salt trough and 12 m^2^ artificial shade (shading with 85% coverage). The *Panicum maximum* cv. Mombasa was established in the area for two years and kept by continuous grazing at 60 cm of residue mass before the beginning of the experiment. A fixed stocking rate of 3.6 AU/ha (animal unit, corresponding to 450 kg BW), that corresponds to an approximated 810 kg/paddock (2 cows). The put-and-take stocking method was used to adjust the stocking rate of each paddock and the additional animal was subject to the same diets as the cows. Forage accumulation was measured every 14 days within two exclusion cages per paddock; the cuts and calculations were performed according to Terra [14], with the adaptation of 14 days between cuts.

To determine the forage intake, grass samples were collected using the technique of hand-plucking, according Prohmann [15]. Data collection was conducted for a period of 40 min, starting at 6:00 a.m. and therefore before the morning feeding. In these collections, animals were followed at less than 2 m to observe their grazing habit and the preference for structural components of the forage. Thus, simultaneously and synchronously with the cows, four samples were collected manually (10 min/sample) of the forage that was being selected and eaten by the animals. The material was stored in a freezer until chemical analysis. The forage:concentrate ratio (For:Con) was determined per animal and calculated based on forage and concentrate intake. The rumen degradable protein balance and rumen undegradable protein balance were calculated using the NRC model application [16] based on concentrate and pasture intake.

The apparent digestibility of diets (grass and concentrate) was determined by the intake of forage and concentrate and fecal output. The fecal output of the cows was estimated using the external marker titanium dioxide (TiO_2_). Thus, on the 6th to 14th day of each experimental period, 10 g of titanium dioxide was placed in the concentrate. At the 11th, 12th, 13th, and 14th day of each trial period, 50-g samples of feces were collected directly from the rectum at 8.00 a.m. and 5.00 p.m. The samples were frozen and later analyzed for titanium concentrations [17]. The forage intake was estimated using insoluble acid detergent fiber (ADFi) as an internal marker. For this, samples of grass, concentrate, and feces were incubated in the rumen of a fistulated cow (this cow did not participate in the trial but received the same diet as treatment with 0.6% of BW of concentrate) with rumen cannula for 288 h and then washed with water and an acidic detergent solution [18].

At the end of each collection period, the pasture, concentrate, and fecal samples were pre-dried in a forced-ventilation oven at 65 °C for 72 h, ground in a Wiley mill through a 1 mm mesh sieve, and homogenized to make composite samples for each animal for each time period. Dry matter (DM), crude protein (CP), neutral detergent fiber (NDF), acid detergent fiber (ADF), and mineral matter (MM) were determined by AOAC [19]. The total carbohydrates (TC) were estimated by the equation proposed by Sniffen [20], and non-fibrous carbohydrates (NFC) were calculated according to the equation proposed by Hall [21].

The blood samples, taken on the 11th day of each period, were taken directly from the tail vein, after milking, using vacutainer tubes containing two drops of heparin to prevent blood clotting. Samples were immediately centrifuged, and the plasma frozen for later analysis of glucose levels and plasma urea, using colorimetric methods and commercial kits.

The urine collection, as “spot” samples, was made on the 11th, 12th, 13th, and 14th day of each experimental period by spontaneous urination. Urine samples were diluted 1:10 (*v*/*v*) with 0.036 N sulfuric acid and then frozen [22]. The concentrations of creatinine and urea were later determined using Labtest and Gold Analisa commercial kits and spectrophotometry to calculate the urinary output [23]. The loss of urea in urine was expressed in mg/d and mg/kg BW.

Cows were weighed at 14 d intervals after milking in the morning. The cows were milked, without the presence of calves, using mechanical milking, at 6:00 a.m. and 18:00 p.m. and the milk weighed daily. To facilitate the milking, 0.2 mL oxytocin was administered via the mammary vein, using a 2.5 mm-diameter needle. Immediately after milking, the calves were released with their mothers for 30 min to maintain the emotional bond and so they could take the residual milk. Milk consumption by calves was determined by weight before and after suckling. The calves were kept with the cows in the pasture, so there would be an interaction between mother and offspring, and to ensure lactogenesis and the persistence of lactation. However, the calves did not have access to the udder, as they were isolated in a shelter, laterally protected with a metal screen.

Milk samples were collected on the 12th and 13th day of each trial period, placed in sterile plastic containers, and kept refrigerated at 4 °C. The composition of the milk was assessed by an ultrasonic method to determine fat, protein, lactose, and non-fat solid contents. The milk yield was corrected to 4% of fat by ECM = (0.432 × kg of milk) + (0.1623 × kg milk × % fat) [24], and the milk yield efficiency was calculated by kg of ECM/kg of DM intake. The presence of subclinical mastitis was evaluated weekly by the California mastitis test (CMT), no cow had mastitis.

The data underwent preliminary analysis with the statistical software R, version 3.0.2 [25]. The data were submitted to the shapiro.test(x) functions to verify the normality of data and the bartlett.test(x) to test homogeneity between variances, of which both functions belong to the stats library of R. After preliminary analyses, regression studies were performed, considering the effects of the model via the procedure lm(x) of the stats library [26].

## 3. Results and Discussion

The production of forage biomass in the paddock was 448.22 kg. The forage accumulation and forage accumulation rate were 896.45 kg DM/ha and 64.28 kg DM/d, respectively. The percentages of leaf, stem, and dead forage mass were 62.95 ± 1.39%, 24.09 ± 0.98%, and 13.35 ± 0.81%, respectively. The chemical composition of *Panicum maximum* cv. Mombasa was: 29.24 ± 0.50% of DM, 4.18 ± 0.10% of CP; 77.19 ± 0.38% of NDF, 43.81 ± 0.32% of ADF, 5.02 ± 0.17% of non-fiber carbohydrate, and 12.35 ± 0.12% of ash.

The DM intake (kg/d and % BW) decreased linearly with the reduction of concentrate levels in the diet (Table 1). With the reduction in concentrate levels, a linear increase in forage consumption was observed, changing the For:Con ratio, demonstrating that the animals had the ability to compensate for the reduction in the supply of concentrate by increasing the consumption of forage. Decreased concentrate levels increased the NDF dietary content by increasing the For:Con ratio; but NDF intake never changed as was expected. The DM intake decreased, indicating that NDF may have acted as a limiting factor in DM consumption [27]. In this study, NDF intake ranged from 1.46% to 1.57% BW (data not presented), indicating that Pantaneira cows have a high capacity for ingesting NDF, and consequently, are animals indicated for organic production, which makes less use of concentrates in the diet [2]. The CP intake decreased with the reduction in concentrate levels, indicating that the concentrate provided a higher proportion of CP than pasture.

The study of digestibility is important to quantify the utilization of feed by the animal; an influence of the concentrate on the digestion of nutrients was observed in this study (Table 2). The digestibility of DM, CP, TC, NFC, EE, MM, and TND showed a decreasing linear effect. However, there was no effect on the concentration of digestible energy. In assessing levels of supplementation for grazing dairy cows, Pimentel [28] found an increase in digestibility with an increase in concentrate offered. The digestibility of NDF and ADF increased with decreasing concentrate in the diet. Ruminal NDF digestibility is typically depressed when ruminal pH is decreased [27]. High ingestion of feeds rich in starch, leading to a higher rate of ruminal fermentation, thus reduces the pH and affects fibrolytic microbial activity [29]. Decreased ingestion of feeds rich in starch, at a low level of concentrate supplementation, can reduce the total amount of short-chain fatty acids (SCFAs) and reduce the proportion of propionate with a consequent increase in the proportion of acetate, by the increase in ruminal NDF digestion [27].

The plasma glucose concentration was not influenced by the percentage of concentrate (Table 3), with observed values considered to be normal for cattle and varying from 53.4 to 72.1 mg/dL [30]. The TC and sugars are converted to SCFAs in the rumen. As glucose is a key nutrient required for milk synthesis and the maintenance of other body tissues, there must be de novo synthesis of glucose in cattle because of activation of the gluconeogenic metabolic pathway in the liver [31]. As a result of this mechanism, the effects of treatments on plasma glucose concentrations are normally not expected.

The concentration of nitrogen urea in plasma (NUP) in mg/dL decreased linearly with a reduction in the percentage of concentrate. For treatments in which 1.2, 0.9, and 0.6% BW of concentrate were used, high levels of NUP were observed. Only in the treatment without concentrate and with the lowest level (0.3% BW) of concentrate supply were NUP concentrations within the range considered adequate for lactating cows: between 7 and 23.5 mg/dL [32]. Rufino Junior [7], testing the effect of different hays with the same For:Con ratio on Pantaneira heifers, observed plasma urea concentrations of 6.71 mg/dL, lower than found in this work. Rennó [23] found levels of 14.54 mg/dL of urea in the blood plasma of Holstein cattle. NUP is a sensitive and fast indicator of RDP balance; its increase may indicate an excess of protein in the diet [33]. However, the RDP balance (Table 1) indicates only the cows from treatment with 1.2% BW of concentrate had a positive balance. The estimated values may have errors because the model does not specifically predict for the Pantaneira breed. Diets with readily fermentable CHO, slowly degradable N sources, and highly negative rumen nitrogen balance, can reduce microbial crude protein synthesis [34]. The high level of NUP associated with RDP balance can indicate that Pantaneira cattle require lower levels of protein in the diet. The urinary excretion of nitrogen was not affected by supplementation of animals as such as found by Agle et al. [35]; the excess of NUP was probably recycled by the urea cycle or secreted in milk.

The milk yield was negatively affected by the reduction in the percentage of concentrate in the diet (Table 4). This behavior was expected given that, in animals for which the energy–protein requirements are partially met, there is a reduced selectivity during grazing: the less digestible fractions of forage are eaten, with negative effects on milk production and the genetic potential of the animal not being reached. Responses in milk yield primarily reflect changes in net energy lactation intake due to changes in DM intake [27]. However, the production of milk corrected for energy was not affected by the reduction in concentrate levels in the diet. Thus, the milk production of animals fed only on grazing was equivalent to those of animals receiving a greater quantity of concentrate (1.2% BW).

The percentages of protein, lactose, and non-fat solids in milk decreased linearly with the reduction in concentrate levels in the diet. According to Krolow et al. [36], lactose concentration of cow milk has little dietary-dependent variability. However, in this study, the lactose content decreased linearly with the decrease of concentrate supplementation level. The low intake of starch can decrease the rumen propionate production and intestine absorption [27], consequently reducing the glucose synthesis by gluconeogenic metabolism and subsequent uptake by the mammary gland for production of lactose [31]. Additionally, the highly negative RDP balance can reduce the number of glucogenic amino acids, which are glucose precursors [37]. On the other hand, the fat concentration in milk increased linearly. Feeding diets high in forage are associated with increased acetate production [38]. Increased molar proportions of acetate, the major precursor of de novo synthesis of milk fat, is consistent with the increased concentration of milk fat [27]. 

The feed efficiency was not affected by reduced levels of concentrate, indicating that the dependence of the milk yield on concentrate is less significant in dairy cows with lower commercial screening, such as the Pantaneira breed. There are good possibilities for the better integration of local animals and the use of grazing areas to the benefit of organic systems [39].

## 4. Conclusions

The reduced levels of concentrate in the diet of Pantaneira cows reduced the dry matter and nutrient intake and altered the digestibility of nutrients, but had no effect on corrected milk yield and milk yield efficiency. Pantaneiro cattle have the genetic potential for the maintenance of competitive production and quality in organic systems.

## Figures and Tables

**Table 1:** Dry matter (DM), forage (For), concentrate (Con), and nutrient intake of Pantaneira cow breed, fed with different levels of concentrate.

Intake	Level of Concentrate (% of Body Weight)					SEM	*p*-Value	
	1.2	0.9	0.6	0.3	0.0		Lin	Qua
DM, kg/d	10.19	9.66	9.12	8.58	8.09	0.304	<0.01	0.78
DM, % BW	2.37	2.28	2.18	2.08	1.99	0.063	0.03	0.63
For, kg/d	5.07	5.82	6.58	7.33	8.09	0.310	<0.01	0.85
Con, kg/d	5.13	3.84	2.55	1.25	0.00	0.264	<0.01	0.53
For:Con Rate	50:50	60:40	72:28	85:15	100:00			
CP, kg/d	1.43	1.13	0.83	0.54	0.25	0.060	<0.01	0.98
NDF, kg/d	6.29	6.30	6.33	6.35	6.39	0.213	0.88	0.81
ADF, kg/d	3.30	3.39	3.48	3.57	3.68	0.128	0.27	0.84
RDP balance, g/d	104	−86	−251	−405	698			
RUP balance, g/d	91	−2	−280	−593	1175			

SEM: standard error mean; CP: crude protein; NDF: neutral detergent fiber; ADF: acid detergent fiber; RDP balance: rumen degradable protein balance [16]; RUP balance: rumen undegradable protein balance [16].

**Table 2:** Apparent digestibility of dry matter (DM) and nutrients of Pantaneira cow breed, fed with different levels of concentrate.

Digestibility	Level of Concentrate (% of Body Weight)					SEM	*p*-Value	
	1.2	0.9	0.6	0.3	0.0		Lin	Qua
DM, %	93.20	93.55	91.23	86.26	78.63	0.868	<0.01	<0.01
CP, %	79.95	78.69	70.69	55.95	34.45	2.49	<0.01	<0.01
EE, %	91.85	88.72	85.00	80.71	75.82	1.091	<0.01	0.49
NDF, %	67.04	68.65	70.14	71.50	72.73	0.563	<0.01	0.81
ADF, %	51.30	52.89	54.52	56.04	57.44	0.443	<0.01	0.69
TC, %	72.73	70.89	68.00	64.09	59.13	0.769	<0.01	0.01
NFC, %	89.56	87.13	84.44	81.47	78.25	0.696	<0.01	0.48
TND, %	70.81	68.77	66.30	63.39	60.03	0.626	<0.01	0.20
DE, Kcal	2.94	2.91	2.89	2.89	2.90	0.027	0.65	0.74

SEM: standard error mean; CP: crude protein; NDF: neutral detergent fiber; ADF: acid detergent fiber; TC: total carbohydrate; NFC: non fiber carbohydrate; EE: ether extract; TND: total nutrient digestible; DE: digestible energy.

**Table 3:** Glucose and urea levels in blood plasma and loss of urinary nitrogen of Pantaneira cow breed, fed with different levels of concentrate.

Plasma and Urine Content	Level of Concentrate (% of Body Weight)					SEM	*p*-Value	
	1.2	0.9	0.6	0.3	0.0		Lin	Qua
Glucose, mg/dL	56.67	56.56	56.44	56.33	56.22	1.600	0.92	0.47
NUP, mg/dL	31.80	28.78	25.75	22.73	19.71	1.133	<0.01	0.81
LUU, g/d	154.43	149.28	144.14	138.99	133.85	7.520	0.29	0.71
LUU, mg/kg BW	366.99	358.19	349.40	340.60	331.81	20.521	0.49	0.99

SEM: standard error mean; NUP: nitrogen ureic in plasma; LUU: loss of urea in urine.

**Table 4:** Milk yield and composition of Pantaneira cow breed, fed with different levels of concentrate.

Milk Yield and Composition	Level of Concentrate (% of Body Weight)					SEM	*p*-Value	
	1.2	0.9	0.6	0.3	0.0		Lin	Qua
Milk Yield, Kg/d	8.24	7.94	7.655	7.40	7.18	0.231	0.04	0.9
ECM, kg/d	8.43	8.26	8.10	7.93	7.78	0.975	0.45	0.58
Protein, %	3.87	3.85	3.82	3.80	3.77	0.017	0.03	0.18
Fat, %	3.93	4.09	4.26	4.39	4.52	0.070	<0.01	0.41
Lactose, %	6.32	6.27	6.22	6.17	6.17	0.025	<0.01	0.26
Not-fat solids, %	11.02	10.93	10.81	10.76	10.68	0.046	<0.01	0.36
MYE, kg/kg of DMI	0.83	0.86	0.89	0.92	0.96	0.109	0.20	0.95

SEM: standard error mean; ECM: energy corrected milk (to 4% of fat); MYE: milk yield efficiency (kg of ECM/kg of DM intake); DMI: dry matter intake.
